# Evaluating the impact of the ‘Four Pest-Free Villages’ program on mosquito-borne disease control in Zhejiang Province, China: a cross-sectional study on knowledge, attitudes, and practices

**DOI:** 10.1186/s40249-026-01422-z

**Published:** 2026-03-04

**Authors:** Jing Ni, Qiyuan Chen, Hui Zhou, Wenrong Zhang, Jinna Wang, Jimin Sun, Zhenyu Gong

**Affiliations:** 1 School of Public Health, Hangzhou Medical College, Hangzhou, 310053 China; 2 Pujiang Center for Disease Control and Prevention, Pujiang, 322200 China; 3 Longyou Centre for Disease Control and Prevention, Quzhou, 324400 China; 4 Department of Communicable Disease Control and Prevention, Zhejiang Provincial Center for Disease Control and Prevention, Hangzhou, 310051 China

**Keywords:** Four Pest-Free Village (mosquito, Fly, Cockroach, and rodent), China, Mosquito-borne disease, Knowledge, attitude, practice, Neglected tropical diseases, One Health, Integrated vector management

## Abstract

**Background:**

Mosquito-borne diseases (MBDs) pose a persistent public health threat globally. The "Four Pest-Free Villages" program, which targets mosquitoes, flies, cockroaches, and rats, has been in place in Zhejiang Province, China, for more than 9 years. It was recently improved to version 4.0 as a crucial tactic for long-term vector management.

**Methods:**

This cross-sectional study, which included eight "Four Pests Control Villages" and eight matching control villages, was carried out in 2024 in 16 villages spread across five cities in Zhejiang Province. To evaluate knowledge, attitude, and practice (KAP) about infectious diseases spread by mosquitoes, a stratified-cluster random sampling technique was used. In every dimension, KAP ratings of 70% or greater were deemed satisfactory. Chi-square tests and multivariate logistic regression analysis were used to find influential factors (α = 0.05). Ten stakeholders, including community managers, public health specialists, and resident representatives, participated in in-depth, semi-structured interviews to further examine the project’s efficacy and overall worth.

**Results:**

Residents of the "Four Pest-Free Village" (which targets mosquito, fly, cockroach, and rodents) showed significantly higher levels of knowledge (*P* < 0.01), attitudes (*P* < 0.05), and preventive practices (*P* < 0.01) regarding mosquito-borne diseases compared to control villages, according to 1447 valid questionnaires. While older age [odds ratio (*OR*) = 1.7–1.9], higher education (*OR* = 2.2), and minority status (*OR* = 2.4, all *P* < 0.05) predicted better knowledge and attitudes within “Four Pest-Free Villages”, younger residents, migrant workers, and individual farmers showed higher levels of preventive practice (*P* < 0.05) in control villages; gender effects on practice varied between sites.

**Conclusion:**

The “Four Pest-Free Village” program—targeting mosquito, fly, cockroach, and rodents—significantly improved residents’ health literacy and their adoption of vector control practices. This underscores the necessity of customized interventions based on demographic variables and the significance of integrating health education programs with environmental management. The results offer useful recommendations for maximizing vector control initiatives and enhancing public health outcomes in both urban and rural regions. To further improve and bolster these tactics, future studies should incorporate more variables and broaden the data sources.

**Graphical Abstract:**

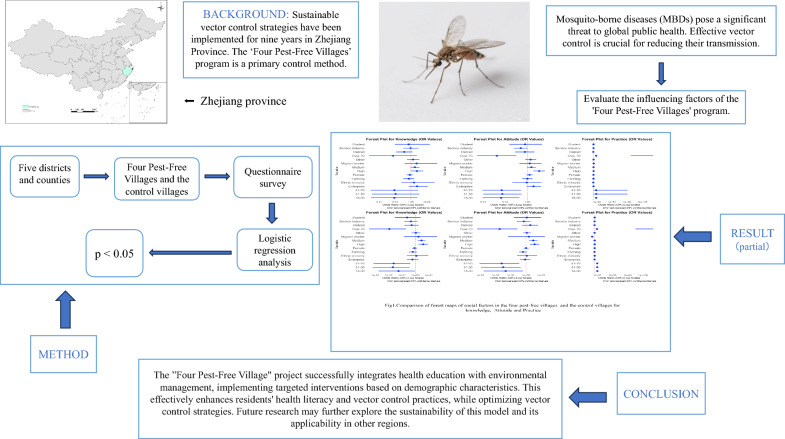

**Supplementary Information:**

The online version contains supplementary material available at 10.1186/s40249-026-01422-z.

## Background

Vector-borne diseases pose a hazard to more than 80% of the world's population, with mosquito-borne diseases (MBDs) making up the entire area [1, 2]. Mosquitoes transmit some of the most dangerous pathogens, such as *Plasmodium* (the causative agent of malaria) and various viruses [3, 4]. Mosquitoes are the principal vectors for malaria parasites, while *Aedes and Culex* species are primarily responsible for transmitting arboviruses [5]. *Aedes* mosquitoes (such as *Aedes aegypti and Ae. albopictus*) are major carriers of dengue virus, Zika virus, yellow fever virus, chikungunya virus, and West Nile virus, leading to diseases like dengue fever, Zika fever, and yellow fever [6–8]. *Culex* mosquitoes (notably *Culex tritaeniorhynchus*) primarily transmit Japanese encephalitis virus and West Nile virus, and are also vectors for filariasis, which can lead to Japanese encephalitis outbreaks and lymphatic system disorders [9]. Newly developing infections pose the danger of extraordinary epidemics, even though several of these diseases have long been public health issues. According to the 2024 World Malaria Report [10], there were an estimated 263 million malaria cases and 597,000 malaria-related deaths globally in 2023. This indicates that there were almost 11 million more cases in 2023 than in 2022, but the death toll was essentially unchanged. The case fatality rate of dengue fever, which is spread by mosquitoes, is similar to that of malaria. [11–13]. Dengue epidemics surged in a number of nations in 2023, and the World Health Organization (WHO) classified them as a Level 3 emergency [14].

MBDs, as a group of common tropical diseases worldwide, have a high degree of infectivity [15, 16]. Approximately half of the population worldwide now faces the risk of contracting dengue fever, with an estimated 100–400 million infections occurring each year [14]. Over 400,000 deaths occur each year, while yellow fever, chikungunya, and Zika have casued severe outbreaks in many urban areas areas [17]. It is estimated that by the year 2050, approximately half of the global population will be at risk of transmission of vector-borne viruses [18]. The primary method for alleviating the public health burden of most MBDs is based on vector control interventions. The global vector control response 2017–2030 indicates that effective and sustainable vector control should be adaptable to the conditions of various regions around the world [19]. In 2020, the report “Ending the Neglect to Attain the Sustainable Development Goals: A Road Map for Neglected Tropical Diseases 2021–2030” introduced sustainable dengue vector control interventions across ten key endemic countries and established control and monitoring systems in five out of six WHO regions [20]. The primary risk factor for dengue virus transmission is human-mosquito contact, highlighting the importance of reducing mosquito populations and limiting human exposure to mosquitoes [21]. Individuals living in densely populated informal settlements with poor sanitation infrastructure (e.g., open water containers and discarded tires), outdoor workers such as construction workers and street vendors who are active during peak mosquito biting times, and residents of suburban areas with limited vector control programs all face a significantly higher risk of MBDs [22]. A newly emerging challenge is the climate-driven expansion of mosquitoes into temperate regions, as rising temperatures extend the mosquito activity season and increase transmission potential [23].

One specific manifestation of the global vector control response 2017–2030 in China is the creation of mosquito-free villages [24], serving as a sustainable vector control measure primarily implemented in Zhejiang [25–27]. The aim is to reduce the density of vectors through environmentally friendly treatment methods, thereby decreasing the incidence of vector-borne diseases and achieving health for humans, animals, and the environment. Four Pest-Free Villages in Zhejiang Province exemplify the implementation of “sustainable vector control measures” and the establishment of “participatory and mobilized communities”. The concept of “Four Pest-Free Villages” has been implemented for nearly 10 years, and it is an environmentally friendly model that integrates science popularization, exchange, learning, training, and economic development. Following the comprehensive prevention principle of "environmental governance as the foundation, physical and biological control as the main methods, and chemical control as a supplement", measures for controlling mosquitoes have been referenced in accordance with the GB/T 27775 "Guidelines for integrated vector management—Urban" and T/ZJPCA 001 "Guide of sustainable control and prevention of vectors in rural areas—the four pests". This research investigates differences in knowledge, attitudes, and practices related to MBDs among residents of the Four Pest-Free Villages compared to the control villages. It also examines how fostering a participatory and mobilized community enhances mosquito awareness and lowers mosquito densities in the Four Pest-Free Villages, intending to provide recommendations for further improving and developing these communities [28]. This will offer valuable references and recommendations for the ongoing optimization and future development of such villages. It aims to provide insights for sustainable vector control measures and integrated vector management, offering a practical basis for the One Health concept [29], and contributing to the timely achievement of the objectives outlined in the 'Global Vector Control Response 2017–2030', specifically to 'reduce the burden and threat of vector-borne diseases impacting humans' and 'lower the incidence and mortality rates of vector-borne diseases worldwide' [23, 30–33].

## Methods

### Study sites

The study was carried out in several villages in five regions of Zhejiang Province. These areas contain all of the province's terrain types: the northeastern alluvial plain, the middle hilly basin, and the southwest mountainous territory. Zhejiang is situated between latitudes 27°02′ and 31°11′ north and longitudes 118°01' and 123°10' east. The yearly precipitation ranges from 1100 to 2000 mm, and the overall climate is categorized as subtropical monsoon climate. The province has different seasons, and the combination of heat and moisture creates ideal circumstances for mosquito breeding.

### Specific measures

The Four Pest-Free Village project has explored practical, eco-friendly methods for controlling vector-borne diseases in rural areas. It relies mainly on environmental modification, physical and biological measures, while chemical interventions are kept to a minimum, and has developed standards and evaluation systems for creating a Standard Village for Mosquito and Vector Control (SVMV). It has also investigated workable, sustainable strategies for managing vector-borne illnesses in rural regions. (i) Environmental modification: During three phases of their life cycle, mosquitoes are submerged. The mosquito population can be successfully decreased by keeping water bodies flowing or removing standing water from mosquito breeding grounds. (ii) Physical measures: Sticky traps or light traps (solar mosquito lamps) are used to eradicate rats, cockroaches, flies, and mosquitoes. (iii) Biological metrics: In the "Four Pest-Free Village," biological management mainly targets mosquitoes by using mosquito-eating fish (or other appropriate biological means) [29].

### Questionnaire survey

Face-to-face interviews with a sampling population from five regions—including "Four Pest-Free Villages" and the control villages—were part of our cross-sectional study, which was published in compliance with the STROBE statement [34]. Below is a description of the sampling strategy.

Sampling frame and participants selection we used a “stratified-cluster random sampling” scheme with partial randomization at the prefecture level.

*Stratification (purposive)*. We first deliberately chose five of the eleven prefecture-level cities in the province—Quzhou, Shaoxing, Jiaxing, Lishui, and Jinhua—so that the study sites covered eastern, northern, western, and central Zhejiang as well as coastal, hilly, and plain topographies due to staff, financial, and local cooperation constraints.

*Cluster randomization*. (1) Within the five cities, we enumerated all 121 “Four Pest-Free Villages” that had been certified by December 2022 (out of 250 villages in the complete administrative list) to form the primary sampling frame. (2) Eight demonstration villages were drawn from this frame in a single step with probability proportional to size (PPS). (3) For each selected demonstration village, we randomly chose one control village from the same frame, matched on “no certification” and similarity in terrain and main economic activity, yielding eight pairs (16 villages in total).

*Within-household selection*. Village household registers served as the secondary sampling frame. Sixty households per village were chosen through simple random sampling; one resident aged 18 or older from each household was then randomly invited. To ensure at least 960 valid interviews, we oversampled by selecting 70–75 households per village to account for anticipated non-response. However, final refusal rates were lower than expected, resulting in 1447 valid questionnaires, all of which were included in the analysis. This increased statistical power and enabled the examination of subgroup effects. No post-hoc replacement or selective inclusion of participants was performed. The purpose of this questionnaire was to obtain information regarding the awareness of core knowledge about MBDs in the study area and participation in vector control activities. The questionnaire consisted of multiple-choice and single-choice questions, printed on an A4 sheet. The first page collected demographic information (such as place of residence/government district, education, occupation, etc.). The single-choice questions covered basic knowledge of MBDs, attitudes towards MBDs and mosquitoes, and daily practice regarding mosquito handling. The multiple-choice questions delved into in-depth knowledge of MBDs and the types of control assistance that respondents hoped to receive related to MBDs. Before this study, the questionnaire was validated through a pre-test to serve as a quality control measure. The overall Cronbach's α for this questionnaire was 0.787. The four-dimensional structure was validated through Exploratory Factor Analysis (EFA), with a cumulative variance contribution of 63.2%. Each item's factor loading was ≥ 0.49, with no significant cross-loadings, indicating that the questionnaire possesses good internal consistency and structural validity. It can be used to assess the mosquito-borne knowledge and influencing factors among residents in Four Pest-Free Villages.

### Expert interviews

To explore the goals and significance of the Four Pest-Free Village projects, we conducted semi-structured expert interviews with 10 stakeholders—6 public health experts, 2 community managers, and 2 local resident representatives—between November and December 2024 [35, 36]. Given field constraints and participant preferences regarding privacy, interviews were not audio-recorded. Instead, three trained researchers attended each interview: one served as the primary note-taker while the other two provided supplementary documentation. All three independently recorded detailed handwritten notes during the session, capturing key statements, contextual observations, and non-verbal cues. Within 24 h, the team synthesized these notes into a single structured, anonymized interview record to enhance accuracy and minimize recall bias. The interview guide (provided as Supplementary Material S1) covered six domains: implementation effectiveness, brand development, patent potential, signage design, cultural symbolism, and economic development potential. The data was analyzed using thematic analysis: one researcher performed initial coding, and emerging themes were refined through team discussion to ensure analytical rigor. All data were stored securely in password-protected digital files and locked physical folders, accessible only to the research team.

### Variables

The main explanatory variables were age, gender, ethnicity, education level, residence, and occupation, while the outcome variables were KAP.

### Statistical analysis

All survey data were entered into a database with Microsoft Excel^®^ software (Microsoft Corporation, Redmond, WA, USA) and analyzed with R 4.4.1 software (R Foundation for Statistical Computing, Vienna, Austria). Given that KAP scores were derived from summed item responses with limited integer ranges, they were treated as non-normally distributed and reported as median (interquartile range).The *χ*^2^ test was employed to analyze count data between groups, and the Bonferroni method was used to adjust the *P*-value in multiple comparisons. Multiple logistic regression was utilized to analyze factors influencing the awareness rate of core knowledge regarding MBDs, with a significance level of α = 0.05. For single-choice questions, the scoring criteria for KAP were: 1 point for a correct answer and 0 points for an incorrect answer; in terms of beliefs, a response of 'very important' or 'very necessary' received 1 point, while any other response received 0 points. For multiple-choice questions, 1 point was awarded for each correct option. For both single-choice and multiple-choice questions, a score reaching 70.0% or above of the total score for that section was considered to indicate the corresponding level of KAP.

### Model performance and external validation

This study aimed to evaluate and compare the predictive performance of models for health-related knowledge, attitudes, and practices (KAP) through the application of receiver operating characteristic (ROC) curve analysis [37]. All validation set curves were clearly above the diagonal, indicating performance significantly better than chance. The knowledge and attitude models showed excellent generalizability, with higher area under the curves (AUCs) in the external validation set (control villages) compared to the training set (intervention villages). The practice model displayed acceptable generalizability, with a slight decline in performance, reflecting the challenges of predicting practice. Best-fit models—based on predictors such as education level (positive association), ethnicity, and age group (negative associations)—were used to predict outcomes in a separate test dataset [38, 39]. This process, consistent with TRIPOD guidelines, provides robust and unbiased model performance estimates (see Fig. 3). For additional related figures concerning the validation set, please see Online Appendix 1.

## Results

### Descriptive results

A total of 1460 questionnaires were collected. Thirteen questionnaires were insufficient for analysis and were therefore excluded, resulting in 1447 fully completed questionnaires.

### Demographic characteristics

There were 857 females (59.2%) and 590 males (40.8%) among the 1447 valid questionnaires. The age distribution was symmetric and unimodal, peaking in the 31–50 age range (558 people, 38.6%), followed by those under 18 (102 people, 7.0%), and those over 70 (169 people, 11.7%). 1429 respondents (98.8%) were of Han ethnicity, whereas 18 respondents (1.2%) belonged to ethnic minorities. Regarding education, 409 (33.9%) had a bachelor's degree or above, 198 (13.7%) had a junior college education, and 759 (52.5%) had just completed high school. Agricultural workers (449 people, 31.0%) and workers in governmental or public institutions (435 people, 30.1%) were the two most prevalent occupations; the remaining occupations are described in Table 1.

**Table 1:** Demographic characteristics of the survey subjects (*n* = 1447)

Demographic characteristics	Number of participants	Composition ratio (%)
Gender		
Male	590	40.8
Female	857	59.2
Age		
< 18 years old	102	7.0
18–30 years old	224	15.5
31–50 years old	558	38.6
51–70 years old	394	27.2
> 70 years old	169	11.7
Ethnic group		
Han ethnicity	1429	98.8
Others	18	1.2
Education		
Elementary school and below	244	16.9
Junior and Senior high school/Vocational high school	515	35.6
Junior college	198	13.7
Bachelor's degree or above	490	33.9
Occupational status		
Government agencies/institutions	435	30.1
Enterprise	94	6.5
Service Industry	68	4.7
Student	113	7.8
Engaging in agriculture	449	31.0
Labor work	23	1.6
Retirement	41	2.8
Others	224	15.5
Whether residing in Four Pest-Free Village		
Yes	793	54.8
No	654	45.2

### Knowledge, attitude, and practice (KAP)

In this study, 1099 residents were aware of knowledge regarding the prevention of MBDs, accounting for 75.0%, while 384 residents were unaware, making up 25.0%. In both the Four Pest-Free Villages and the control villages, the highest awareness rate of basic knowledge about MBDs was for question 6 (Do you regularly clean and sort garbage around your home?), at 94.2%, while the lowest was for question 4 (Is there a vaccine to prevent dengue?), at only 43.3%.

In Four Pest-Free Villages, there were 477 individuals who possessed an attitude towards controlling MBDs, accounting for 60.0%; while 313 individuals did not possess such an attitude, accounting for 40.0%. In the control villages, 334 individuals had an attitude towards controlling MBDs, representing 52.0%; whereas 320 individuals did not possess this attitude, accounting for 48.0%.

In Four Pest-Free Villages, 292 individuals demonstrated preventive practices against MBDs, representing 36.0%; whereas 501 individuals did not engage in such practices, accounting for 64.0%. In the control village, 146 people, or 22.0% of the population, demonstrated MBD preventative practices, whereas 508 people, or 78.0% of the population, did not. The remaining occupations are described in Table 2.

**Table 2:** The current status of knowledge, attitudes, and practice regarding MBDs in Four Pest-Free Villages of Zhejiang Province compared to the control villages

Project	Location	Category	Number of participants	Composition ratio (%)
Knowledge	Four Pest-Free Village	Possess	615	77.0
		Lack	118	23.0
	Control village	Possess	484	74.0
		Lack	170	26.0
Attitudes	Four Pest-Free Village	Possess	477	60.0
		Lack	313	40.0
	Control village	Possess	334	52.0
		Lack	320	48.0
Practice	Four Pest-Free Village	Possess	292	36.0
		Lack	501	64.0
	Control village	Possess	146	22.0
		Lack	508	78.0

**Fig. 1.**
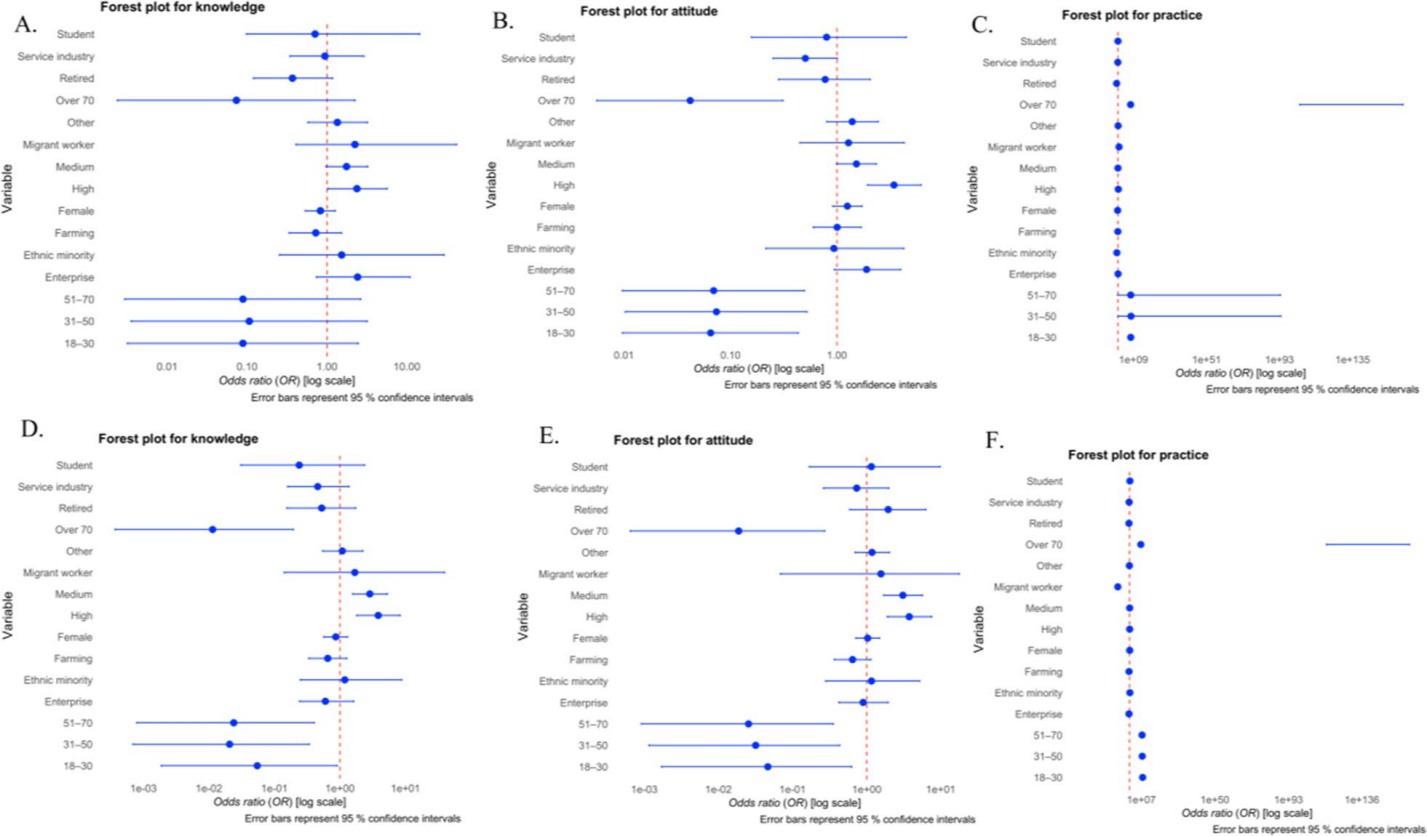
Forest plots for health-related outcomes in the control villages (top row) and the Four Pest-Free Villages (bottom row). **A** Knowledge of the control villages; **B** Attitudes in the control villages; **C** Practices in the control villages; **D** Knowledge in the Four Pest-Free Villages; **E** Attitudes in the Four Pest-Free Villages; **F** Practices in the Four Pest-Free Villages. All models were adjusted for age group, gender, ethnicity, education level, and occupation

Overall, residents of the Four Pest-Free Villages exhibit superior KAP compared to the control villages. The inhabitants of Four Pest-Free Villages exhibit better health awareness, greater health knowledge, and more active participation in health-promoting behaviors. In particular, compared to the control villages, the average knowledge score of the inhabitants in Four Pest-Free Villages is substantially higher (*P* < 0.01). Residents in Four Pest-Free Villages exhibit a stronger positive attitude towards health (*P* < 0.05). Additionally, they show a higher level of participation in health practice (*P* < 0.01). Multivariable analysis showed that although higher educational attainment was significantly associated with greater knowledge and more positive attitudes in both the Four Pest-Free Villages and control villages, its association with preventive practices was observed only in the intervention setting [higher education: adjusted odds ratio (*aOR*) = 1.87, 95% confidence interval (*CI*): 1.08–3.26, *P* < 0.05]. Notably, migrant workers in the intervention villages were nearly five times more likely to adopt recommended practices than those working in public institutions (*aOR* = 4.97, 95% *CI*: 1.70–14.5; *P* < 0.05). In contrast, in the control villages, the *P*-values for the associations of all sociodemographic factors (including education, occupation, age, ethnicity, or gender) with preventive practices were greater than 0.14 (all *P* > 0.14) indicating no statistically significant associations. These findings suggest that the integrated four pest-free intervention not only strengthened the link between knowledge/attitude and practice among the highly educated but also effectively engaged vulnerable occupational groups who are often overlooked in routine public health initiatives (Fig. 1).

**Fig. 2:**
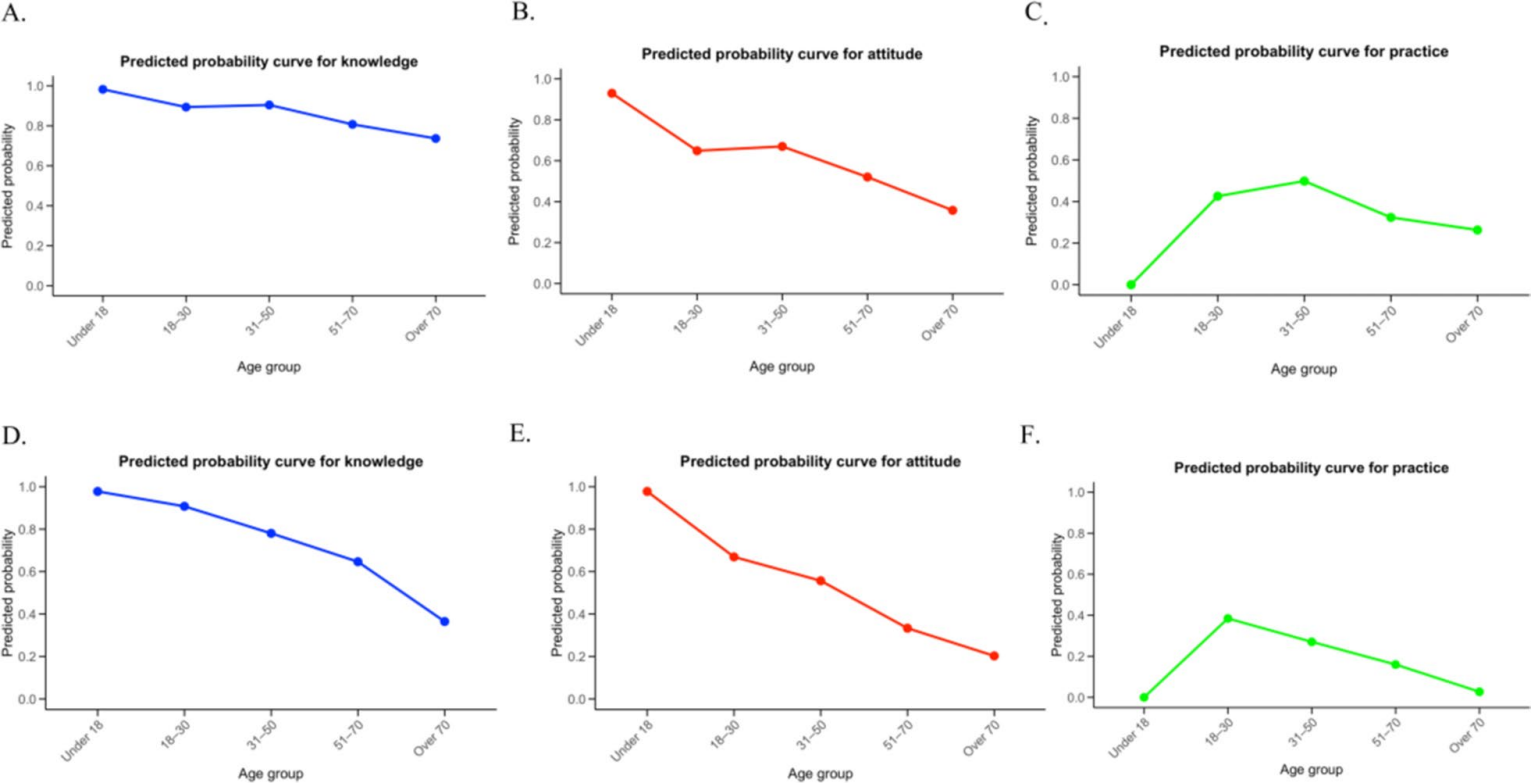
Predicted probability curves for health-related outcomes in the Four Pest-Free Villages (top row) and the control villages (bottom row). **A** Knowledge of the Four Pest-Free Villages; **B** Attitudes in the Four Pest-Free Villages; **C** Practices in the Four Pest-Free Villages; **D** Knowledge in the control villages; **E** Attitudes in the control villages; **F** Practices in the control villages. All models were adjusted for age group, gender, ethnicity, education level, and occupation

**Fig. 3:**
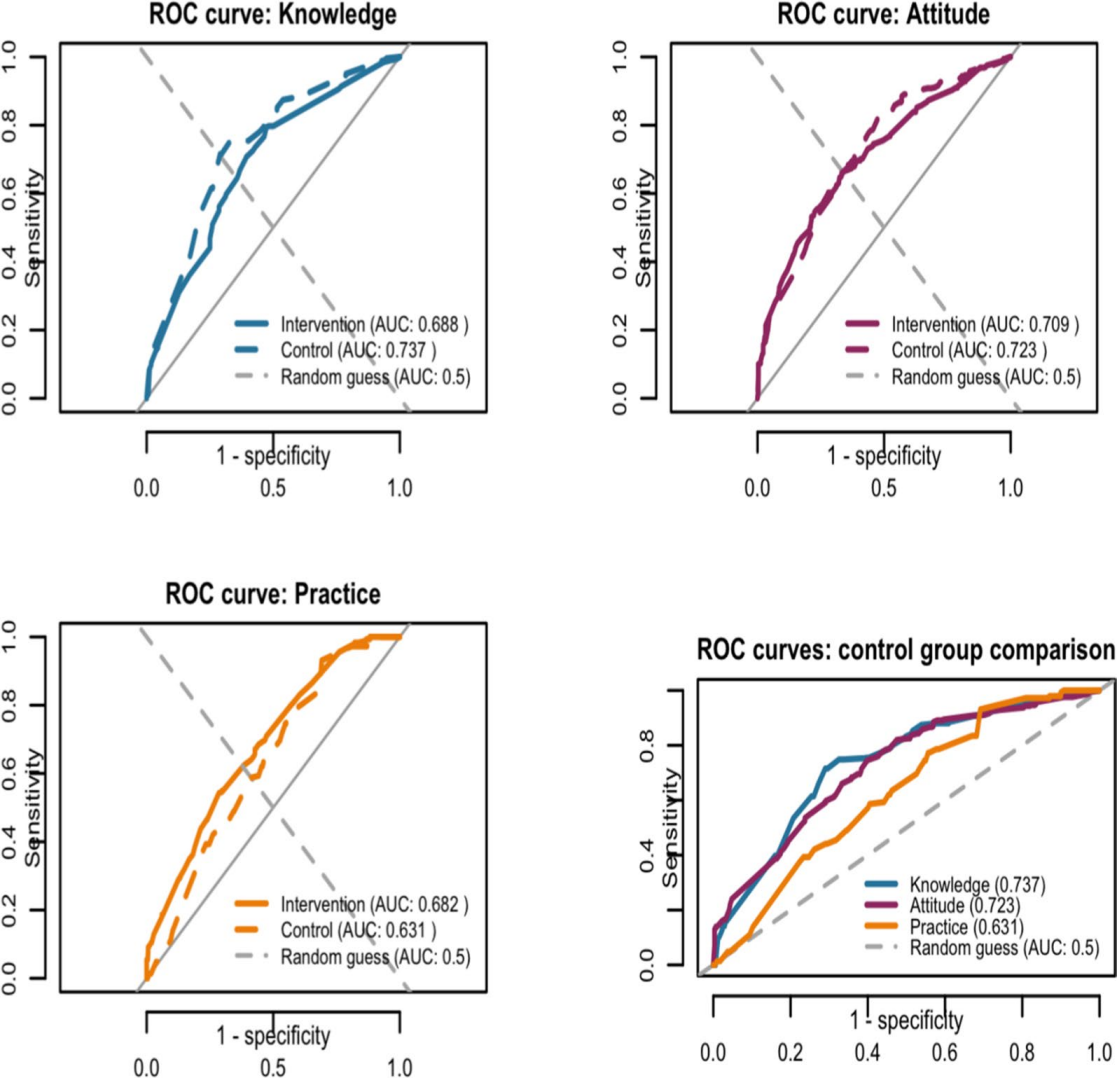
ROC Curves for Model Performance in Training and Test Sets

Through the analysis of the Logistic regression model, both the model for the Four Pest-Free Villages and the control village exhibited good predictive capabilities. (i) Knowledge model: the AUC for the Four Pest-Free Villages was 0.70, while that for the control village was 0.78. (ii) Attitude model: the AUC for the Four Pest-Free Villages was 0.71, and for the control village, it was 0.75. (iii) Practice model: the AUC for the Four Pest-Free Village was 0.69, and for the control village, it was 0.72 (see Fig. 2 and 3).

ROC curves showed model performance in intervention villages (training set, solid lines) and control villages (test set, dashed lines). The curves plot sensitivity versus 1-specificity across classification thresholds, with the AUC representing overall discriminative ability (higher values indicate better class separation). Knowledge and attitude models showed superior generalizability (higher test-set AUC), while the practice model preserved acceptable performance. Specifically, the KAP prediction models trained on data from the intervention villages achieved AUCs of 0.737, 0.723, and 0.631, respectively, when externally validated on an independent sample from the control villages. Notably, the discriminatory performance of the knowledge and attitude models was slightly higher in external validation than in the training set (AUC: 0.688 and 0.709), suggesting good generalizability. Although the AUC for the practice model decreased modestly, it remained well above that of random guessing (AUC: 0.50).

## Discussion

Residents in the intervention villages exhibited markedly higher KAP scores, underscoring the effectiveness of integrated health education and environmental governance strategies [15]. These results represent an outcome assessment of the most recent stage of the "Four Pest-Free Villages" project and demonstrate the effective execution of the WHO Global Vector Control Response 2017–2030 in China. Mosquito density has decreased by almost 80.0% since its start [15], and there have been no locally acquired instances of MBDs for almost 10 years. The model has been scaled across major *Aedes* risk areas nationwide, from Beijing in the north to Hainan in the south, and from Fujian in the east to Chongqing in the west [40], and has drawn over 300 delegations with over 5000 visitors for study tours, creating a nationally recognized public health brand. Additionally, it was presented at the 9th and 10th International Conferences on Sustainable Vector Control.

Our mixed-methods research shows that the program has produced synergistic effects spanning public health, community identity, and rural rejuvenation in addition to decreases in KAP gaps and illness incidence. Expert interviews and KAP surveys regularly revealed significant improvements in population health outcomes and significant decreases in vector-borne diseases like dengue, which is consistent with Zhejiang's most recent provincial consensus on vector control. Experts unanimously agreed that “mosquito-free village construction is not only a cornerstone strategy for preventing dengue and other arboviral diseases but also a comprehensive platform for enhancing quality of life and advancing rural revitalization" during the 2024 Zhejiang Provincial Seminar on Mosquito-Borne Disease Prevention.

Strategic branding has strengthened both public recognition and resident identification with the initiative, while culturally resonant signage and symbols have fostered social cohesion. Notably, the project has stimulated local economic development: county-level CDC officials reported integration with tourism through innovations such as “mosquito-interest museums” and health-themed cultural products. Municipal and county health authorities have proposed expanding the model to “mosquito-free towns” and incorporating it into educational outreach (e.g., school-based study tours) and regional cultural branding. Senior experts emphasized that long-term sustainability hinges on cross-sectoral collaboration—with agriculture, culture-tourism, and publicity departments playing pivotal roles [38]. Collectively, these outcomes illustrate that the “Four Pest-Free Villages” model transcends conventional vector control, functioning as an integrated driver of health promotion, cultural renewal, and rural economic growth.

These broader impacts are grounded in tangible gains in health literacy and environmental quality. The establishment of the Four Pest-Free Villages has significantly enhanced residents’ health-related knowledge and awareness through coordinated health education and environmental management activities [38]. Effective environmental governance provides a healthier living environment, which in turn facilitates the adoption and maintenance of healthy behaviors. These results offer robust empirical support for replicating this approach in similar settings, suggesting that comparable interventions can effectively elevate population health [39].

The study further identified higher KAP levels among elderly residents—a pattern likely attributable to demographic shifts, as out-migration of younger adults has left older individuals as the primary resident group. A key enabler is the initiative’s participatory design, which links community engagement with modest economic incentives. Organizing paid environmental cleanup activities has effectively mobilized older adults, leading to deeper involvement and sustained behavioral change. Moreover, traditional communication channels such as village loudspeaker systems align well with their information-seeking preferences [41]. As a health-vulnerable population, older adults may also exhibit heightened awareness of MBD risks, further reinforcing participation.

Although residents in control villages generally showed lower levels of health-related knowledge (*P* < 0.01) and less positive attitudes toward health (*P* < 0.05) compared to those in Four Pest-Free Villages, younger residents there still demonstrated relatively high engagement in preventive practices [40], which highlighting the need for age-tailored health promotion strategies [42]. For youth, digital platforms—including social media and mobile applications—represent promising avenues for health communication [43]. In contrast, community-based activities and health lectures remain effective for middle-aged and elderly rural residents [44, 45]. Additionally, culturally adapted interventions are warranted for ethnic minority and occupational subgroups to ensure inclusive participation in healthy lifestyles [46].

Despite its contributions, this study has limitations. First, data were collected from only five sites, limiting generalizability to other ecological or socioeconomic contexts. Second, the analytical model did not account for potential confounders such as household socioeconomic status or social network structures [47]. Third, the use of convenience sampling at the city level combined with cluster sampling at the village and household levels may constrain external validity and introduce residual confounding [48]. While observed associations are robust, validation in probability-based, multi-stage random samples is recommended. Future research should expand data sources to incorporate additional contextual variables [49] and adopt longitudinal designs to better capture temporal dynamics in KAP [50].

## Conclusions

This study confirms that the 'Four Pest-Free Village' model effectively enhances health literacy and prevents mosquito-borne infectious diseases through a community-driven approach that deeply integrates health education with environmental management. The institutionalized integration of multi-departmental collaboration (health, agriculture, culture and tourism, etc.) and the incorporation of health promotion into rural regeneration plans constitute its primary innovation rather than technological advancements. This model tackles a crucial issue in the global management of vector-borne diseases: how to accomplish sustainable governance that goes beyond emergency response. It creates a self-reinforcing mechanism for long-term participation by connecting illness prevention with local interests, such as ecotourism, cultural identity, and community empowerment. For low- and middle-income nations dealing with interconnected health, environmental, and development issues, this approach offers a scalable and flexible integrated solution. Such robust, community-led models are becoming increasingly crucial for the security of global health in light of how vector spread is being exacerbated by climate change.

## Supplementary Information

Additional file 1.

Additional file 2.

## Data Availability

The datasets used and analyzed during the current study are available from the corresponding author on reasonable request.

## Abbreviations

MBDs: Mosquito-borne diseases
KAP: Knowledge, attitude, and practice
SVMV: Standard Village for Mosquito and Vector Control
WHO: World Health Organization
AUC: Area under the curve
ROC: Receiver operating characteristic
*OR*: Odds ratio
a*OR*: Adjusted odds ratio
*CI*: Confidence interval
EFA: Exploratory Factor Analysis
