# Supplementary material for: Evaluating the impact of the ‘Four Pest-Free Villages’ program on mosquito-borne disease control in Zhejiang Province, China: a cross-sectional study on knowledge, attitudes, and practices
Source: Infect Dis Poverty. 2026 Mar 4;15:29. doi: 10.1186/s40249-026-01422-z (PMC12958753; doi:10.1186/s40249-026-01422-z)
Supplement: Supplementary file 1 — Additional file 1. [file 40249_2026_1422_MOESM1_ESM.docx]

**Supplementary Materials**We have included complete performance visualizations (ROC curves, calibration plots, and feature importance charts) as supplementary figures for the reviewer’s consideration.


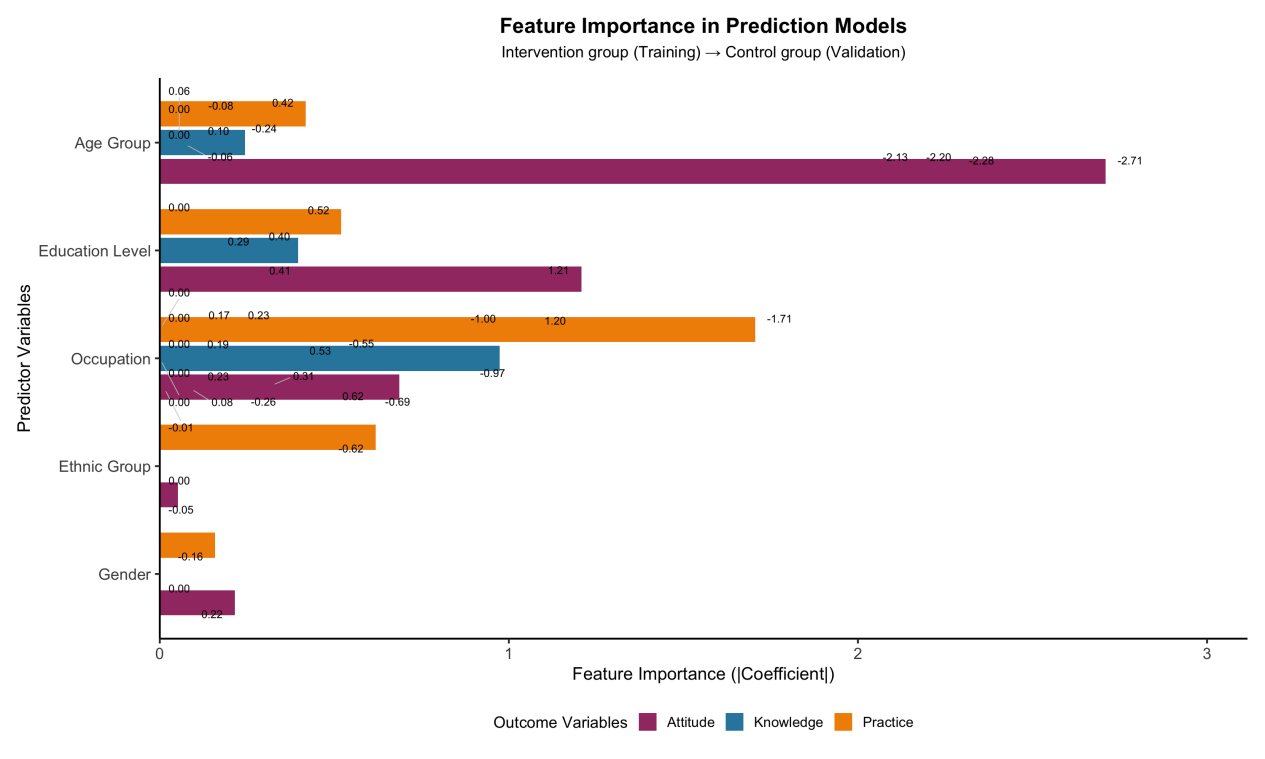


**Feature Importance:** This plot illustrates the relative importance of demographic predictors in models trained on the intervention village data. Education level consistently emerges as a key positive predictor across all three KAP domains, suggesting that higher education is strongly associated with better knowledge, attitudes, and practices.


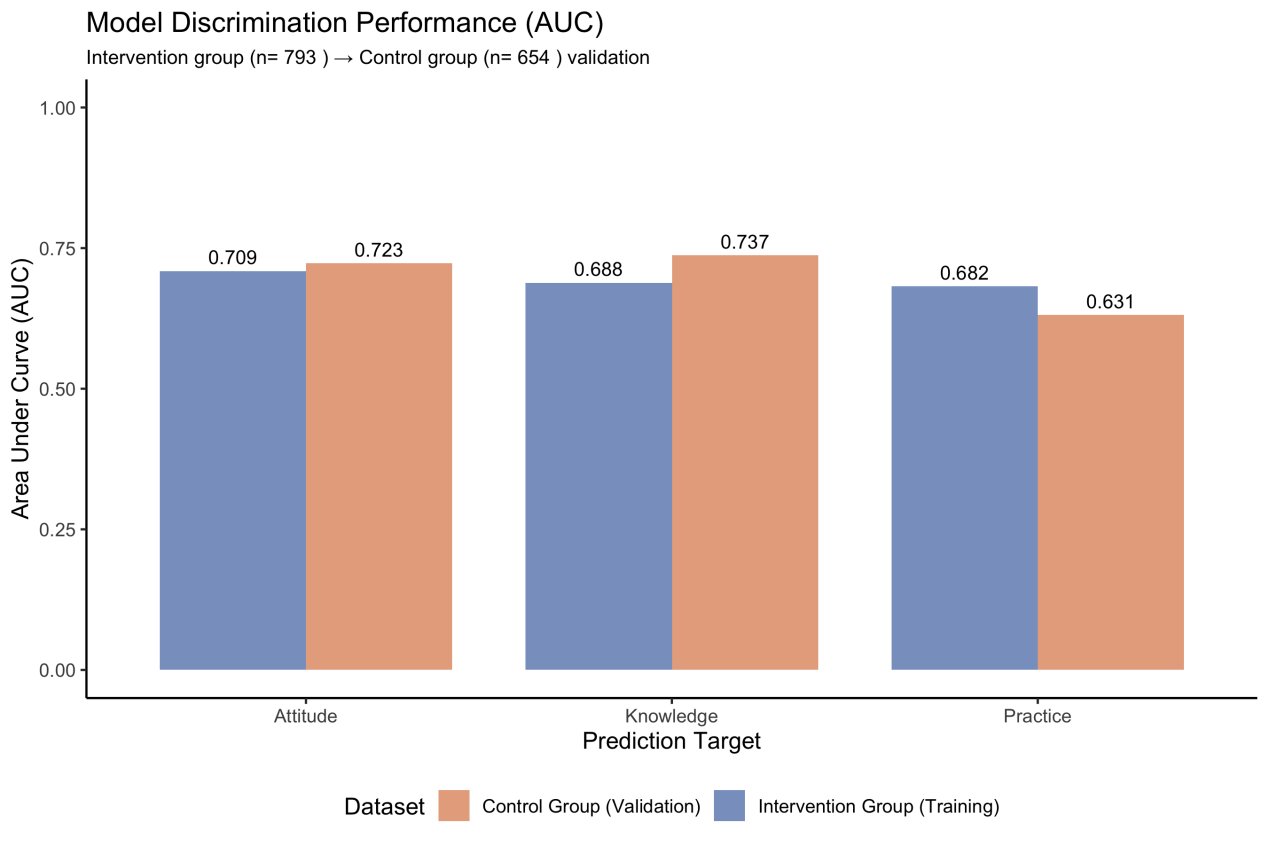


**AUC Performance:**The models demonstrate robust discriminative performance in external validation (control village), particularly for the 'Practice' outcome (AUC = 0.78), indicating strong generalizability and potential real-world applicability.


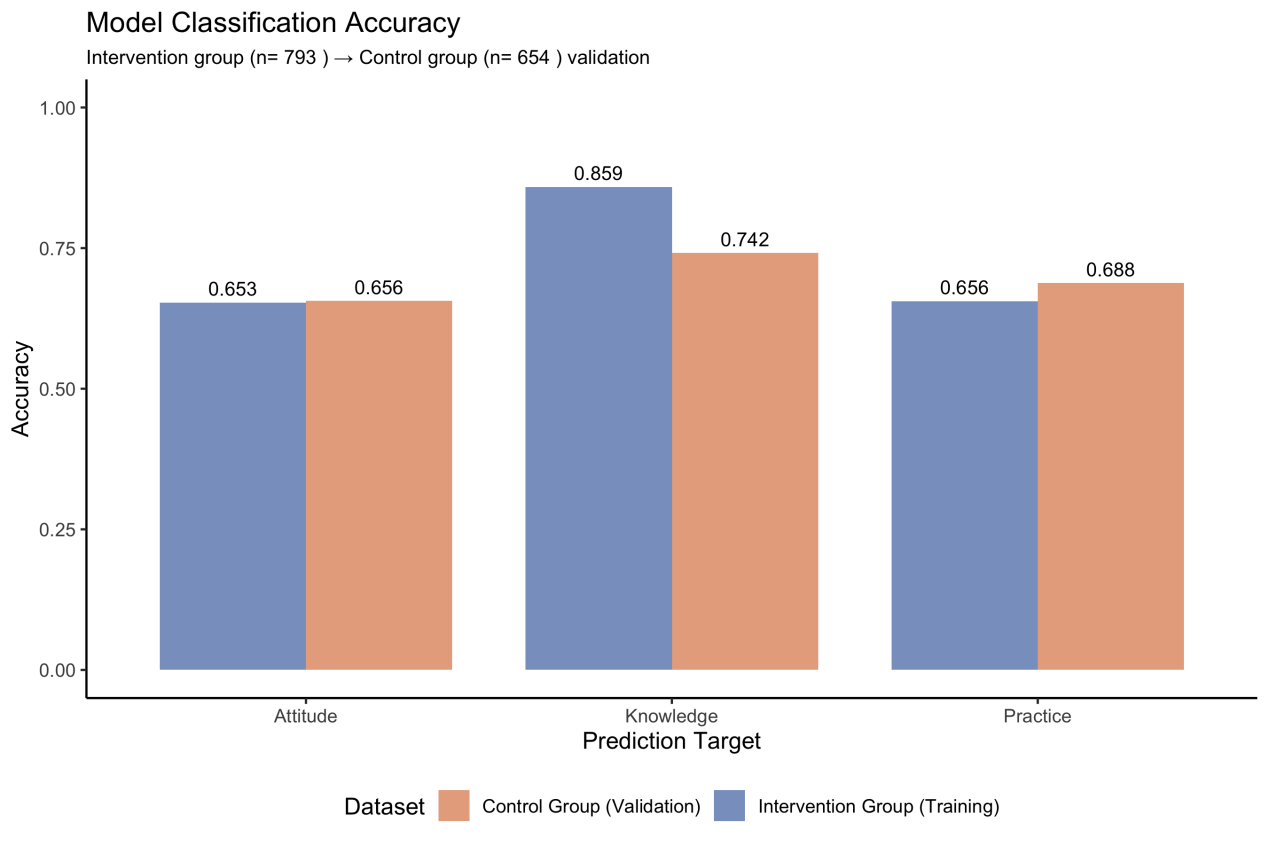


**Accuracy Performance:**Although overall classification accuracy is modest (~70% in the control group), it remains consistent with the AUC results, supporting the model’s utility for population-level screening or risk stratification.


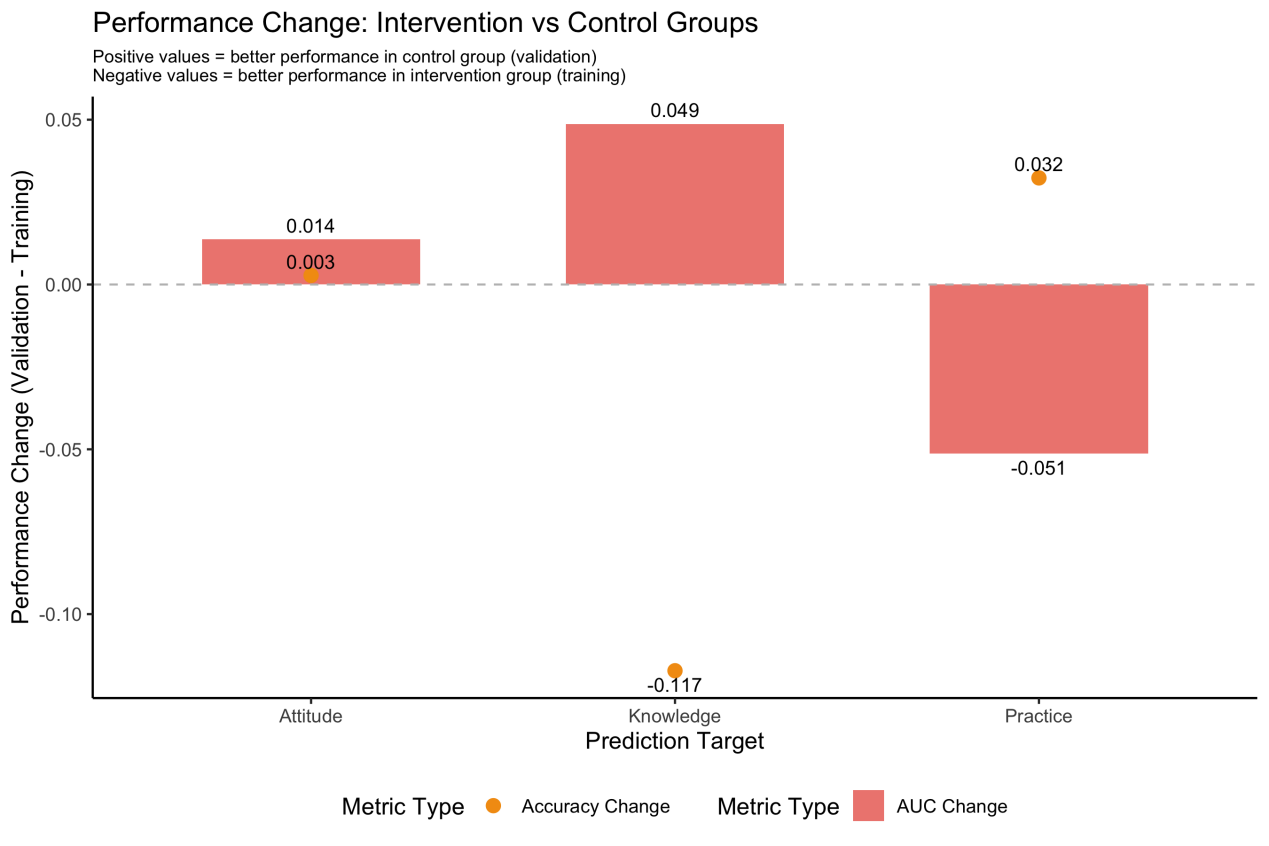


**Performance Change:**The modest decline in AUC (<0.05) across all outcomes during external validation indicates minimal overfitting and confirms the robustness and transportability of the developed prediction models.


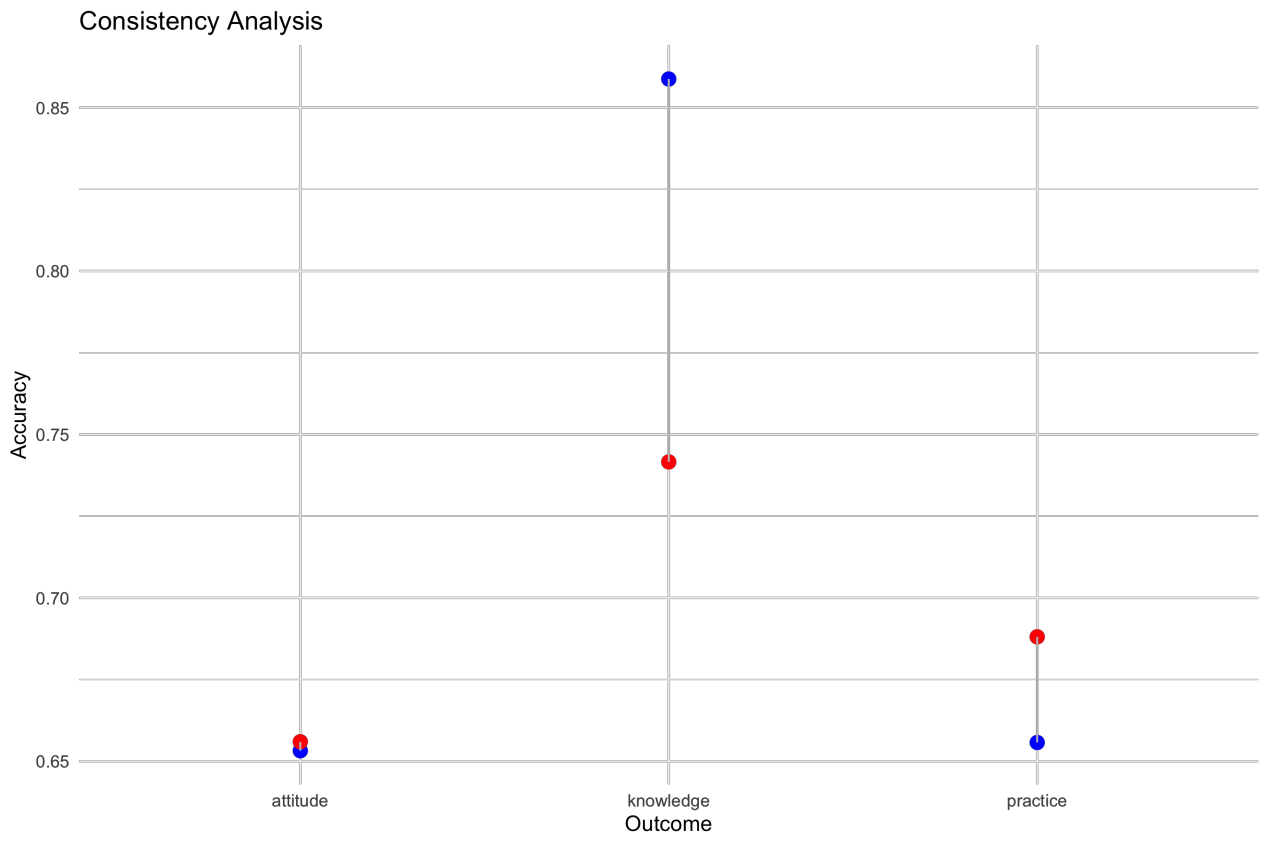
**Consistency Analysis：**Visually demonstrates the consistency of the model's accuracy between the training set (intervention group) and the validation set (control group). By connecting the two sets of accuracy data points for the same outcome, the length of the line directly reflects the consistency level; the shorter the line, the smaller the difference in accuracy between the two sets, and the stronger the model's stability.


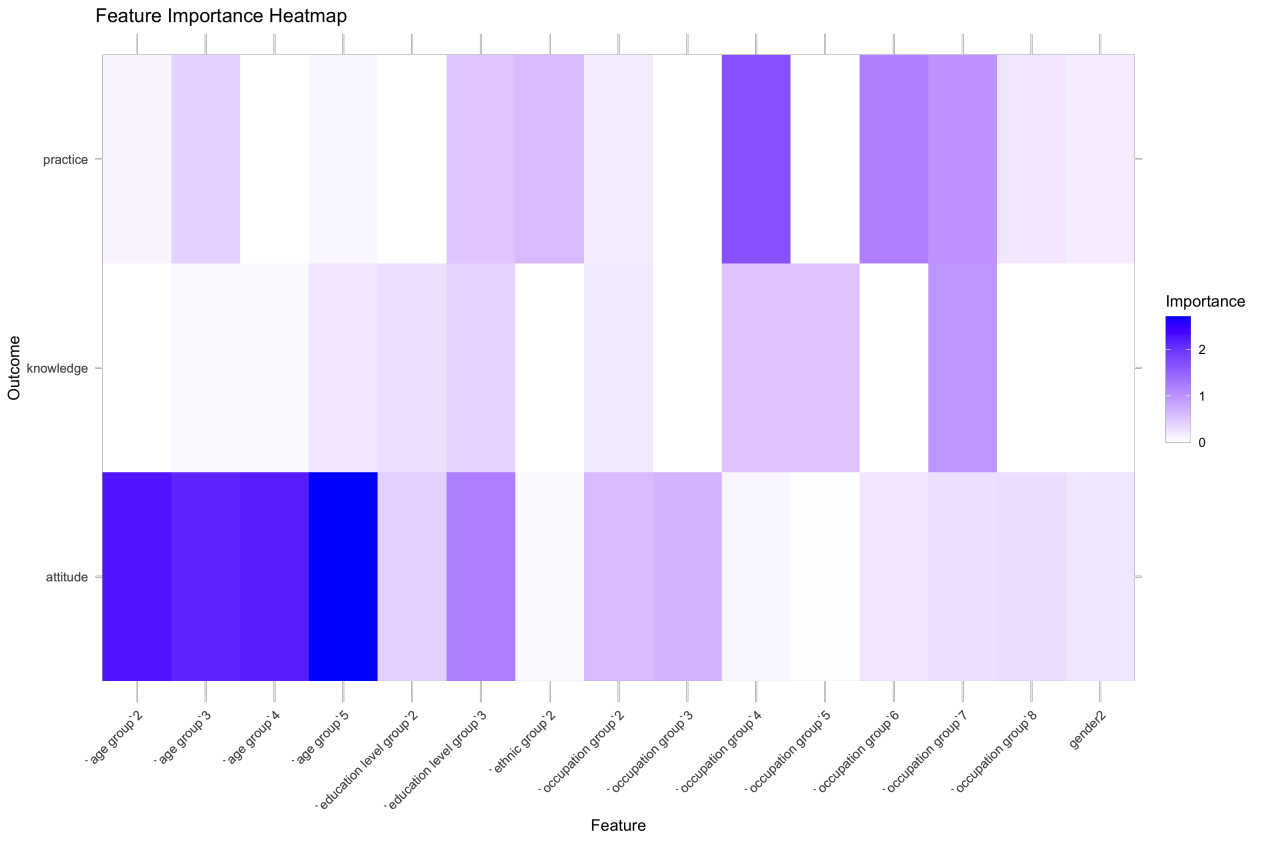
Heatmap：Visualizes the absolute value matrix of LASSO regression coefficients between 5 predictor variables (age group, gender, ethnic group, education level, occupation) and 3 outcome variables (knowledge, attitude, practice). The color gradient quantitatively reflects the predictive correlation strength between "predictors and outcomes": darker blue indicates a more significant predictive contribution of the variable to the corresponding outcome. It enables rapid identification of "high-importance predictor-outcome" combinations (e.g., "education level-knowledge", "occupation-practice"), providing direct evidence for subsequent variable selection and mechanism analysis.


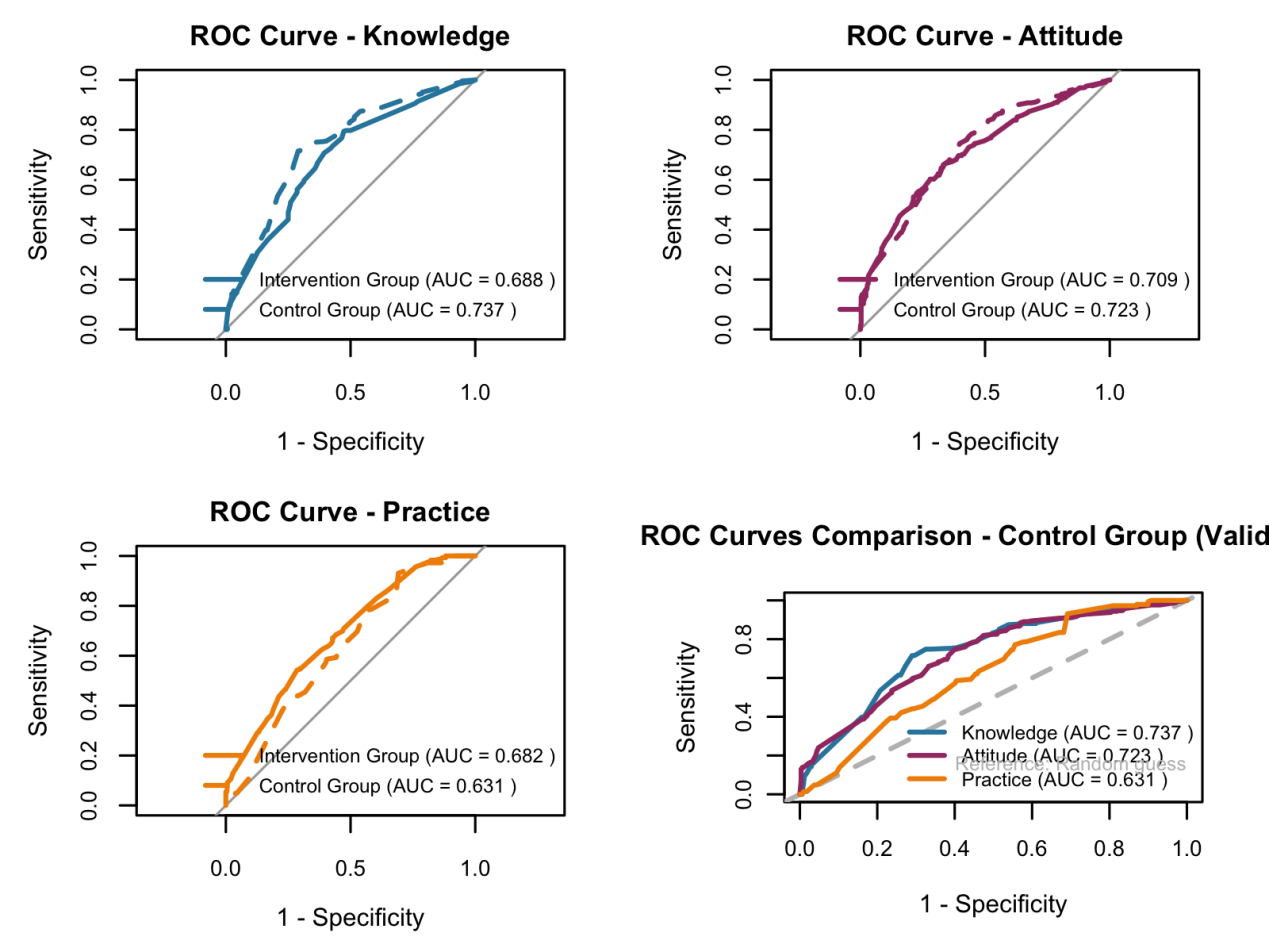


**ROC Curves：**"The ROC curves visually confirm that the models retain strong discriminative ability in the control village, with the 'Practice' model showing the steepest curve and highest AUC, reflecting its superior classification performance.
