# Supplementary material for: Evaluating the impact of the ‘Four Pest-Free Villages’ program on mosquito-borne disease control in Zhejiang Province, China: a cross-sectional study on knowledge, attitudes, and practices
Source: Infect Dis Poverty. 2026 Mar 4;15:29. doi: 10.1186/s40249-026-01422-z (PMC12958753; doi:10.1186/s40249-026-01422-z)
Supplement: Supplementary file 2 — Additional file 2. [file 40249_2026_1422_MOESM2_ESM.docx]

**Four Pests-Free Village Interview Guide**

1. Experience and Involvement : How did you get involved in the “Four Pests-Free Village” initiative? Could you share a moment or experience that stood out to you?
2. Perceived Value : In your view, what’s the most real and tangible benefit of the Four Pests-Free Village for local residents?
3. Challenges: How smoothly has the initiative gone? Have you encountered situations where villagers didn’t quite understand it, or where different departments had trouble coordinating? How were those handled?
4. Reflection on Approach: “Compared to the past, what do you think is the biggest difference with the current Four Pests-Free Village approach?” And do you ever worry that despite all the effort, the results might not last?
5. Suggestions for the Future: If this work continues next year, what’s one thing you’d most like to improve? Or if higher-level authorities could support just one thing, what would you hope for?
